# Challenges faced by healthcare workers at a central hospital in Zimbabwe after contracting COVID-19: An interpretive phenomenological analysis study

**DOI:** 10.4102/safp.v64i1.5428

**Published:** 2022-03-29

**Authors:** Idah Moyo, Avhatakali A. Ndou-Mammbona, Azwihangwisi H. Mavhandu-Mudzusi

**Affiliations:** 1 HIV Services, Population Services International Zimbabwe, Harare, Zimbabwe; 2 Department Health Sciences, College of Human Sciences, University of South Africa, Pretoria, South Africa; 3 Department of Graduate Studies and Research, College of Human Sciences, University of South Africa, Pretoria, South Africa

**Keywords:** COVID-19, healthcare workers, IPA, phenomenology, psychosocial challenges, support system

## Abstract

**Background:**

Healthcare workers play a critical role in the delivery of healthcare services. Because of the high risk of exposure to healthcare workers, the emergence of coronavirus disease 2019 (COVID-19) has had a significant impact as they struggled to contain the pandemic. The purpose of this study was to explore and describe the challenges they faced after contracting COVID-19.

**Methods:**

An interpretative phenomenological analysis (IPA) design was employed to gain insight into the lived experiences of healthcare workers who contracted COVID-19 in the course of their duties. This study involved participants who were healthcare workers based at a central hospital in Bulawayo, Zimbabwe. Data were collected through in-depth interviews that were audio recorded. A sample size of ten was reached based on data saturation.

**Results:**

The study showed that healthcare workers lacked psychosocial support, experienced economic challenges as they incurred diagnostic and treatment costs. The study also found that the healthcare workers experienced stigma and discrimination both at work and in the community. Findings also indicate that healthcare workers did not receive institutional support. The study demonstrated lack of preparedness at the institution evidenced by inadequate testing for COVID-19 and shortage of personal protective equipment.

**Conclusion:**

This study’s findings will be critical for health authorities, programmers and policymakers to facilitate planning and preparedness for pandemics. The researchers recommend setting up a differentiated service delivery support system for healthcare workers to cater for their mental health and well-being and that of their families.

## Introduction

Whilst the severe acute respiratory syndrome coronavirus 2 (SARS-CoV-2) also called the COVID-19 pandemic started as a localised outbreak in Wuhan China, a global view of the disease shows the tremendous impact it has had.^1^ Its impact, even amongst healthcare workers has been significant as the following statistics illustrate: around 10 000 healthcare workers had been infected and 74 are reported to have died in Italy by April 2020. About 10% of all COVID-19 cases globally were amongst healthcare workers by July 2020.^2^ The same report indicated that in 40 African countries that had reported on COVID-19 infections, more than 10 000 healthcare workers had contracted the COVID-19 virus. With reference to Southern Africa, in South Africa, as of August 2020, there had been 27 000 infections and 240 deaths amongst frontline healthcare workers.^2^ This emphasises the importance of protecting healthcare workers.

Healthcare workers facilitate the continuum of care and containment of such pandemics as COVID-19.^3^ Given the fragile nature of our economy and health systems in Zimbabwe, when healthcare providers, in the middle of the pandemic fall sick, it puts further strain on the healthcare system. The experiences and challenges encountered by healthcare workers after contracting COVID-19 have been documented by some scholars^4,5,6^. In Zimbabwe, there are no known qualitative studies that have been conducted to gain an in-depth understanding of the experiences of the frontline healthcare workers who contracted COVID-19 in the line of duty. In fact, quantitative studies have been conducted by some scholars in Zimbabwe.^7,8^ Therefore, this study aimed at building upon the findings of other researchers, that were largely quantitative, by widening the scope to cover healthcare workers who contracted COVID-19. That similar and related epidemics had a negative impact on frontline healthcare workers can be demonstrated in outbreaks such as Ebola virus disease (EVD), SARS and Middle East respiratory syndrome (MERS). From these studies, it was evident that the healthcare workers experienced stigma and discrimination, increased risks of infection and psychological effects.^9,10^

A lot of evidence from research on lived experiences of frontline healthcare workers caring for patients with COVID-19 has largely been in the Europe, Asia and United States.^4,11^ In addition, studies in countries such as Australia and Pakistan have been conducted demonstrating the lived experiences of patients who have been admitted because of COVID-19.^12,13^ However, very few studies have been conducted to explore and describe the real-life experiences of healthcare workers who have contracted COVID-19.

This study’s findings will be critical for policymakers, programme planners, hospital management and frontline healthcare workers in developing innovative strategies of supporting staff during COVID-19 and other pandemics. This study, therefore, explored the experiences of healthcare workers who contracted COVID-19 in a central hospital in Zimbabwe.

## Research methods

### Design

An interpretative phenomenological analysis (IPA) design was employed to gain insight into the lived experiences of healthcare workers who contracted COVID-19. The IPA^14,15^ facilitated an in-depth exploration of narratives detailing the lived experience of suffering from COVID-19. In addition, the approach enables study participants to narrate their stories based on their lived experiences^14,16^ and facilitate the adoption from an insider’s perspective. The researchers were able to explore and ask critical questions on certain aspects mentioned by the study participants to gain an in-depth understanding of their experiences.^14^

Unlike other approaches, the IPA has a three-fold focus: interpretive, double hermeneutic and idiographic nature.^14^ According to Noon,^17^ IPA focuses on examining the lived experience of the individual by drawing from the concepts of phenomenology, hermeneutics and idiography. This approach has been utilised in related studies and demonstrated to be useful in exploring experiences in healthcare settings.^15^ The phenomenological aspect of IPA is that it focuses on how individuals narrate their experiences and perceive them.^15^ These three aspects enable the researcher to make sense of and understand the phenomenon under study and consider the unique experience of each participant and not as compared with all other participants’ experiences.^14,15^ Thus, the researcher arrives at rich descriptions of individual cases. Therefore, IPA has enabled the researchers to have an in-depth understanding of individual healthcare worker’s experiences after contracting COVID-19.

### Study setting and participants

This study participants included nurses and doctors who were healthcare workers based at a central hospital in Bulawayo, Zimbabwe. The hospital hosts one of the major centres designated for the provision of healthcare services for COVID-19 patients in Zimbabwe. In addition, it acts as a referral facility for the management of COVID-19 in the southern region of the country. Those who met the inclusion criteria (nurses and doctors who had contracted COVID-19 and were working at the study setting) and agreed to participate in the study provided their contact details. The study excluded nurses and doctors who though having contracted COVID-19, were not willing to participate in the study, were either sick or still in isolation during the process of the study. To recruit the participants, the centre managers distributed the information sheets about the study and screening questionnaires to potential study participants on behalf of the researchers. The researchers then collected these forms, conducted a follow-up through phone calls and arranged the dates and preferred time for an interview. The study consisted of ten participants, nine of whom were nurses, whilst the remainder was a medical doctor. The ratio of nurses to doctors is normal because the majority of healthcare workers are nurses. The demographic data of the participants is displayed in Table 1.

**TABLE 1:** Demographic data of the study participants.

Participant code	Age range in years	Gender	Profession	Years of experience
Sizi	31–35	Female	Nurse	10
Vusi	36–40	Male	Nurse	10
Lizi	21–25	Female	Nurse	1
Dumo	36–40	Male	Nurse	7
Noma	31–35	Female	Nurse	7
Siboe	36–40	Female	Nurse	13
Muzi	31–35	Male	Medical doctor	8
Nozi	25–30	Female	Nurse	1
Lihle	36–40	Female	Nurse	10
Thina	30–35	Female	Nurse	4

### Data collection

Data collection was guided by an interview guide. The development of the interview guide was based on literature review and study purpose.^4,5,6,9,10,14^ The interview guide questionnaire consisted of semi-structured, open-ended questions, with possible probes for further clarification. In addition, a pilot study was conducted, involving three nurses who were not part of the study participants and who worked in other departments. This process assisted the researchers in refining the interview guide and this led to minor adjustments.

Individual interviews, led by the first author were conducted between January 2021 and March 2021. The interviews lasted for 60 min or more. As a result of the potential risk associated with the spread of COVID-19, all the interviews were conducted virtually, through a cell phone call that was audio recorded. All interviews were conducted in the same language for all participants and no translations were required. An interview guide guided the interview process. Before the interview was conducted, the researcher informed the participants that the questions would focus on their experiences after contracting COVID-19.

### Data analysis

All audio-recorded interview data were transcribed verbatim into written text. To gain an in-depth understanding of the participants’ lived experiences, framework for analysis IPA was used to analyse the transcripts.^14^ According to Noon,^18^ IPA focuses on examining the lived experience of the individual by drawing from the concepts of phenomenology, hermeneutics and idiography. This approach has been utilised in related studies and demonstrated to be useful in exploring experiences in healthcare settings.^15^ The three researchers analysed the transcripts independently using an IPA framework.^14^ An academic, who was not part of the study and is an expert in IPA, was requested to act as an independent co-coder, given audio recordings and transcripts to compare. Each researcher read the transcript several times and listened to the audio recording a few times. The following steps, as outlined by Smith and Osborn,^14^ were followed: (1) reading and re-reading the transcript; (2) note taking and developing emergent themes; (3) clustering the emergent themes; (4) crafting a master table of themes composed of superordinate themes, subthemes and extracts from the interviews; (5) examining and comparing the similarities between the master tables of the themes and (6) compiling a single master list composed of a superordinate theme, themes and subthemes. Thereafter, an independent coder was given a table of themes from all researchers to check and compare. Following this process, the research team then met to compare and discuss the preliminary list of superordinate and subordinate themes and develop a detailed description of their meaning. The team had a consensus on discussion and agreed on the final master table, composed of superordinate themes, subthemes and associated excerpts from the transcripts (Table 2).^14,16^ All this was done to ensure credibility.

**TABLE 2:** Superordinate themes, themes and subthemes.

Superordinate themes	Themes	Subthemes
Psychosocial impact of COVID-19 on healthcare workers	Support system	Institutional support
		Peer and family support
		Unavailability of a transport support system
	Psychological effects	Immediate reaction after testing positive to COVID-19
		Anxiety and fear
	Stigma and discrimination	Work-related stigma
		Community stigma
Finance-related challenges	Healthcare-related costs	Treatment costs
		Indirect costs
Recommendations from study participants	Staff preparedness	Testing of frontline healthcare workers
		Securing adequate PPE for frontline healthcare workers
	Psychosocial support system	Counselling
		Follow-up activities
		Health worker welfare

PPE, personal protective equipment.

### Trustworthiness

Several steps were taken to ensure the credibility and trustworthiness of all analysis detailed in this study. Firstly, it is important to recognise that experiences of participants are understood through the subjective interpretation of the researcher.^19^ The first author who collected data is experienced in qualitative research. In addition, the researcher did a lot of introspection and internal examination to explore personal feelings, experiences and biases and all these were bracketed so as to enhance objectivity. This approach allows one to become less assuming about another’s experience, to be open, compassionate and non-judgemental and present data from the perspectives of the study participant, thus ensuring credibility.^16^ In addition, peer debriefing was carried out following every interview and irregularities identified were addressed. An independent co-coder experienced in qualitative research (who is outside the context of the study) listened to the audio-recorded interviews, reviewed and assessed transcripts to review the emerging and final categories from those transcripts and the final themes or findings of a given study. A consensus was reached on the final master table of themes. This afforded the interviewers the opportunity for continued self-reflection throughout the entire process. By coding and recoding many times, comparing the themes and categories with a co-coder, researchers ensured dependability. To enhance authenticity, verbatim extracts from the interviews were utilised. In addition, the research team also met to discuss and compare the initial findings and agreed on super and subordinate themes.

### Ethical considerations

Protecting the rights of the participants is considered a significant ethical issue^18^. To adhere to the study protocol, informed consent documents and research instruments were reviewed and approved by the University of South Africa’s College of Human Sciences Research Ethics (NHREC registration numer: Rec-240816-052) and the Medical Research Council of Zimbabwe (MRCZ/A/268) before the commencement of the study. Permission to conduct the study was also obtained from the Ministry of Health and Child Care through the hospital chief executive officer. All the participants gave a verbal consent before beginning to participate in the study. As part of the consent process, participants were informed that participation was voluntary and that the interviews would be audio recorded. Participants were also informed that they were free to decline or discontinue participation at any time, if they wish so. Participants were assured, both verbally and in writing that data would be handled confidentially and that the results would be reported in such a way that identification of the informants would be impossible. All identifiers were removed from the transcripts, pseudonyms were utilised to enhance anonymity and the data were stored securely on a password-protected computer. The study participants were provided with a detailed information sheet that explained the details of the study and they gave an informed consent after assimilation of essential information.

## Results

The themes that emerged from data analysis are presented in Table 2. The results are presented according to superordinate themes, themes, and subthemes. Three superordinate themes, namely (1) psychosocial impact of COVID-19 on healthcare workers, (2) finance related challenges and (3) recommendations from study participants are outlined.

### Psychosocial impact of COVID-19 on healthcare workers

This superordinate theme focuses on the psychosocial impact of COVID-19 healthcare workers who contracted the virus. It is composed of three themes as follows: support system, psychological effects and stigma and discrimination.

#### Support system

The participants indicated that the support system was found lacking and is discussed under the following subthemes: lack of institutional support, family and peer support and lack of organised transport for healthcare workers.

**Institutional support:** It emerged that some members of staff felt unappreciated because there was no follow up to check if they were recovering or had challenges. When they reported for duty, no communication was initiated to explore how they were coping. Some participants appreciated the research interview and felt that they have had an opportunity to talk about their experiences after contracting COVID-19. During the interview, participants also expressed the fact that they would have appreciated a message via a text or phone call as an acknowledgement that the system cares about them as individuals. One participant also stated that:

‘I never got any support from the hospital management. No one even checks how me, and my family were coping and being on isolation. I only got two phone calls from the hospital, the first one telling me that my COVID-19 test results were positive, the second one telling me that I had stayed off duty for 4 weeks and was deemed negative hence I needed to report for duty. When I got back the Matron who attended to me indicated that they apologise for not being able to do a follow-up during time of isolation.’ (Nozi)

‘In terms of institutional support, I never got any, not even a phone call, WhatsApp or text message to check if I had recovered or not and whether how I was coping psychologically. The very day I was done with isolation, I got a call that I had a duty and isolation was over. I would have appreciated at least some communication from my workplace to check on me. I feel relieved and happy that today I have had to share my experiences after contracting COVID-19.’ (Noma)

**Peer and family support:** Frontline workers who suffered from COVID-19 got physical care and emotional support from their family members. However, participants felt that their families were very anxious and would have benefited from some form of support or counselling [*expert supp ort*]. Participants in this study also got support from colleagues, which was mostly virtual:

‘The support that I got was from my colleagues from the same ward, because around 10 to 18 nurses in the same ward had tested positive. So as a group we supported each other.’ (Thina)

‘My colleagues would communicate mostly through WhatsApp to check on me how I was feeling. A WhatsApp group of those that got infected was formed, hence we supported each other, sharing advice for example on home remedies. We also prayed together.’ (Lizi)

‘My husband was so understanding and was my pillar of strength. I got a lot of support even from my siblings as well. I used to talk to my brothers and sisters. They would phone every day encouraging me, so the support was more from my family.’ (Sizi)

‘’Whilst I got a lot of support from my family, they had a lot of anxieties about my sickness. I wished there was some way of counselling them, or getting some form of support from professional experts or from my workplace.’ (Muzi)

**Unavail ability of a transport support system:** When frontline healthcare workers tested positive to COVID-19 whilst at work, they used public transport back home. One participant indicated that she was traumatised by feelings of guilt as she was afraid that she would transmit the virus to other passengers:

‘After getting my positive COVID-19 test results, I had to find my way home using public transport. I had no choice since I did not have other means of transport. However, I felt guilty by exposing other passengers to the virus.’ (Lihle)

#### Psychological effects

The provision of psychological support to healthcare workers after contracting COVID-19 is critical in enhancing their mental well-being. This theme is about the psychological effects that were experienced by study participants on receipt of the COVID-19 test results.

**Immediate reaction after testing positive to COVID-19:** In this study, frontline healthcare workers who contracted COVID-19 reported how at first, they were in denial and felt traumatised on receipt of their COVID-19 positive test results. They were also filled with anxiety, disorientation and fear for themselves and their families. Participants had this to say:

‘When the results came out, they were positive. It hit me hard. It was difficult to believe or to accept that I had COVID-19. I really became scared. I initially felt the results were not true. The moment was traumatic and frustrating.’ (Sboe)

‘I felt overwhelmed and frustrated. I asked myself several questions ‘why me? Who else have I passed it on to? The period was a difficult one.’ (Lihle)

**Anxiety and fear:** In this study, it was found that frontline healthcare workers who contracted COVID-19 were full of anxiety and fear whilst they were waiting for COVID-19 test results. The post-test positive COVID-19 test results period was also characterised by the same anxiety and fear. Participants had this to say:

‘I was anxious and had this fear that I would die because some of the patients that had suffered from COVID-19 turned out to be bad, with some dying. I had this fear of dying and or spreading the disease to my family. I never thought I would recover.’ (Lizi)

‘During the period I was waiting for results I was very anxious. My major fear and anxieties were that I was going to die and leave my child. I was worried I had infected members of the family, in particular my mother-in-law who has diabetes and hypertension and so was more vulnerable to covid.’ (Siboe)

Participants in this study expressed their frustration because of the uncertainty brought about the novel corona virus. The perception on uncertainty emanated from the number of new cases and deaths that were being reported from social media and talks from people around. The following excerpts demonstrate those emotions:

‘After listening to the news, I would be ever frustrated, not knowing what tomorrow brings for me, whether I would be in a better or worse condition. I was afraid of what the future held for me.’ (Thina)

#### Stigma and discrimination

Participants in this study experienced stigma and discrimination both at work and in the community.

**Work-related stigma:** The study found that healthcare workers who had contracted COVID-19 were stigmatised by their colleagues on return to work as narrated by Vusi:

‘Most people would run away from me and I would say, I am now negative. They would say how do we know that you are negative when you did not test? It was not a good experience for me. I experienced some stigma & discrimination, if you go to a place where people are grouped, you see them disappearing one by one.’ (Vusi)

**Community stigma:** Participants in this study who had contracted COVID-19 indicated that they experienced stigma from the neighbours and landlords. One participant was requested to vacate the accommodation after the landlord discovered that she was sick from COVID-19. Nozi had this to say:

‘After seeing the members of the rapid response team coming to my place, the neighbours and my landlord felt I should move out. I could not stand the comments from the landlord. Despite being sick, I had to look for another accommodation and I left the place. It was a painful experience.’ (Nozi)

### Finance-related challenges

This superordinate theme highlights the finance-related challenges encountered by study participants after contracting COVID-19. The related theme focuses on health-related costs.

#### Health-related costs

This theme demonstrated how the COVID-19 brought health-related costs after the healthcare workers contracted COVID-19. Two themes emerged: treatment and indirect costs.

**Treatment costs:** Whilst participants were battling with illness because of COVID-19, they still had to spend money for medical purposes. Participants had this to say:

‘At that moment it was so stressful, I don’t want to lie, especially looking for finances when I was also too sick. I had to have a COVID-19 test [*PCR*] and chest x-ray at a private clinic using my own finances. My medical aid could not cover these costs.’ (Lihle)

‘It was a difficult and painful experience for the family. There were a lot of expenses, for example, I also had to purchase medicines at my own expense.’ (Noma)

It also emerged that there were hidden costs associated with contracting COVID-19.

**Indi rect costs:** Whilst it was difficult to quantify the individual costs that healthcare workers incurred after contracting COVID-19, the following extracts show that there were expenses involved:

‘I paid for my own transport. My husband required transport to bring food for me whilst I was admitted.’ (Lizi)

‘I had to use home remedies. These also needed to be brought. Also, the fact that I was sick with no appetite, meant that different foodstuffs had to be bought at a cost to the family.’ (Dumo)

The participants were requested to make recommendations on how to respond to this and other pandemics. The following superordinate theme emerged.

### Recommendations from study participants

This superordinate theme highlights the recommendations that were made by study participants. It is composed of two themes, namely staff preparedness and psychosocial support systems.

#### Staff preparedness

The research participants indicated that there was lack of preparedness within the institution evidenced by inability to provide consistent testing for COVID-19 and inadequacy of PPE. The theme has two subthemes: testing of frontline healthcare workers and securing adequate PPE. The participants made the following recommendations as shown here:

**Testing of frontline healthcare workers:** ‘Testing frontline healthcare workers for COVID-19 should be prioritised. The hospital management should provide test kits for all frontline healthcare workers.’ (Siboe)

‘The frontline healthcare workers should be tested regularly. I worked from 4 to 5 months with no testing. The testing should be free and carried out fortnightly.’ (Thina)

**Securing adequate PPE for frontline healthcare workers:** ‘The hospital authorities in collaboration with the central government should have a clear policy and strategy on the procurement and distribution of PPE in hospitals during such pandemics as COVID-19.’ (Dumo)

‘Provision of adequate PPE for healthcare workers to prevent reuse of PPE.’ (Sizi)

#### Psychosocial support system

The theme focuses on recommendations that were made towards provision of the psychosocial support. The recommendations were classified under the following subthemes: counselling support, follow-up activities and healthcare worker welfare.

**Counselling support:** ‘I would recommend that there should be counsellors who are designated to provide counselling for frontline workers all the time whether they have COVID-19 or not because it is traumatic to go through the experience or to suspect that you have COVID-19.’ (Vusi)

‘Institutions should be innovative. For example, there is need for the formation of support groups for champions and people newly infected with the virus. The role of the support group would be to share the lived experiences and provide counselling support to each.’ (Noma)

**Follow-up activities:** ‘There is need for more engagement with the sick healthcare workers over the phone for example, to encourage the worker and make him or her feel more appreciated and supported.’ (Muzi)

‘After discharge there should be a follow up to find out about how the recovery is going. I am home right now, and no one is making a follow up except my sister in charge. All healthcare workers have suffered from COVID-19 at one time or another so whether negative or not we have been affected even mentally.’ (Sizi)

**Healthcare wo rker welfare:** ‘Government should intervene and take care of healthcare workers. If a healthcare worker runs out of money government should provide food hampers and medication.’ (Nozi)

‘If the nurses could have their own buses than mixing with public, it could be better. Even if someone has tested positive to COVID-19, they get into the same bus. That increases the spread of the virus.’ (Thina)

‘There should be shorter working hours for healthcare workers to reduce the risk of exposure and facilitate periods of rest.’ (Vusi)

## Discussion

This study found that some healthcare workers who play a pivotal role in the healthcare system contracted COVID-19. As result of this, they suffered psychosocial and finance-related challenges, particularly those who experienced severe symptoms. The discussion will focus on the following key findings and their implications: the experienced challenges and recommendation that were made by study participants.

In terms of support, the healthcare providers were supported by family and peers. Some had prayer networks. This is consistent with studies in Uganda and Ghana where nurses used prayer as a coping strategy.^9,20^ These preceding studies established that prayer triggered resilience and mitigated stress in healthcare providers.

This study found a lack of support for healthcare workers that contracted COVID-19 by the health institution. Other studies, emphasise the importance of psychological support for the mental well-being of healthcare providers. Another study^21^ observed the need for organised psychiatrists and psychologists to provide mental health support for frontline healthcare workers during COVID-19. The researchers noticed how countries such as United Kingdom, France, Denmark and Malta provide remote counselling sessions with psychiatrists and psychologists. Countries such as Bulgaria, France and Israel have set up helpline through which frontline healthcare providers can be provided with psychological support for stress management and burnout. The researchers feel that unlike in the past where healthcare workers could rely solely on the family for support, fears of contracting the virus have changed those dynamics, thus calling for professional, institution and organised support.^22^ In another study,^23^ scholars call for differentiated psychosocial support to empower healthcare providers.

This study found that on receipt of the COVID-19 positive test result, the participants were stressed. This concurred with study’s findings on the psychological impact of COVID-19 on healthcare providers in Ghana.^24^ The Iranian study^25^ found that the healthcare providers were in a state of helplessness and becoming powerless during the early phase of the pandemic. In this study, frontline healthcare workers who contracted COVID-19 reported how at first, they were in denial and felt traumatised. They were also filled with anxiety, disorientation and fear for their families. A study in Jordan found similar reactions and feelings.^26^ In a related study in Singapore,^5^ the authors call for more attention to the psychological and mental well-being of the frontline healthcare workers during the pandemic. They advocate, as we do, for social support that can relieve them of psychological pressure, promote mental health and eliminate psychological barriers to it. It is our view that peer support and counselling services can go a long way in promoting the mental health of healthcare workers. The Singaporean study^5^ further calls for psychological guidance, mental health lectures and psychological counselling for healthcare workers. These can help promote and maintain a stable mind necessary to deal with the unexpected and reduce anxiety and depression. In this study, participants felt they lacked institutional psychological support. Yet, as a Chinese study^26^ observes, managers should understand the difficulties their subordinates encountered in their professional and social lives and give timely advice and thereby reduce stress. Similar studies had found that support for frontline healthcare workers had come from their families.^27^ Other participants indicated that they relied on peers and prayers as a coping strategy. This was also the finding of studies in Sierra Leone and Uganda.^9,20^ They found that in Ghana, prayer amongst frontline healthcare providers was a foundation for resilience and a source to mitigate stress^24^. Furthermore, a United Kingdom study on support for frontline healthcare workers during pandemics^28^ also indicated that peer support played a critical role in enhancing the psychosocial well-being of frontline healthcare workers affected by COVID-19.

A significant number of participants in this study experienced discrimination or stigmatisation of one sort or the other, both at work and in their communities. Several studies endorse the global nature of stigma and discrimination of healthcare workers. The study found that some participants on returning from isolation or treatment were shunned by workmates. This was consistent with study’s findings in Nigeria that found that healthcare workers who had contracted COVID-19 experienced stigma and discrimination.^29^ Findings by Mostafa^6^ are that Egyptian healthcare workers experienced stigmatisation. Social ostracism including being evicted from rented accommodation faced by some participants was also observed in another study.^30^ Some participants who had contracted COVID-19 reported how their children were shunned by their playmates in a manner similar to what some scholars^31^ termed secondary or associative stigma. They argued that each time an epidemic occurred, incidences of stigma and discrimination were reported.

A study in Sierra Leone^10^ found that several healthcare workers experienced stigma and loneliness during Ebola and SARS epidemics. Recommendations from other studies with regards to work-related stigma is that pre-emptive action should be taken by mental healthcare professionals to disseminate appropriate information about the mode of transmission and type of contacts. This was done so that the discrimination of colleagues is minimised.^32^ A systematic review^33^ of the SARS-CoV-2 pandemic demonstrated that a supportive work environment can be motivating for staff under pressure. Attaching mental healthcare professionals in every hospital and provision of a supportive environment was found to be key in enhancing the mental health well-being.^32^ The World Health Organization^1^ has come out with recommendations concerning stigma and discrimination, these include community awareness and open dialogues to de-stigmatise healthcare providers^34^ and call for effective protection of healthcare workers in and out of workplace.

Some scholars^35^ have argued that in the United Kingdom and United States healthcare workers had a three-fold risk of testing COVID-19 positive. A newspaper article^36^ reported that about 10% of confirmed COVID-19 cases were frontline healthcare workers in Zimbabwe. Unfortunately, the study found that when some staff displayed COVID-19 symptoms and needed to go for a COVID-19 test and get chest X-ray, they did so at their own expense at a time when the economy is strained and the public service workers feel underpaid, with medical aids not meeting the costs for one reason or the other. In addition, if the worker tested positive for COVID-19, they would foot the bill for COVID-19 related medicines. Commenting on the subject of out-of-pocket expenses, another author,^37^ posited that families should be protected from high out-of-pocket costs that affect the financial well-being of frontline healthcare workers. As the frontline healthcare workers would have contracted the virus in the course of duty, the employer should meet the cost. Although the public servants were allegedly insured against COVID-19 by Old Mutual the reimbursement via the parent ministry often took time as it is cumbersome to claim. At the time of the study, none of the participants had received any reimbursement. The researchers and authors of this manuscript recommend that government or health institutions offer free treatment for frontline healthcare workers affected by COVID-19.

When healthcare workers were asked for some suggestions on how to improve the system with regard to healthcare workers who have contracted COVID-19, the following were some of their recommendations:

The study participants recommended that management should follow up on members of staff who are sick individually and make logical arrangements of food and medicines purchasing as staff would be on isolation. This resonates with recommendation by some scholars^38,39^ who argue that nurse managers should know their subordinates and their challenges and keep in regular touch with them. The researchers are of the opinion that because of the pandemic and the number of healthcare workers involved, this work would need to be assigned to one or two managers tasked with making a virtual follow up and support and giving feedback to the rest of management. The researchers feel that the task is feasible and attainable and would enable the management to provide an effective support system including assistance in purchase of food, medicine and planning (for those with no relative to carry out the task because they would be in isolation). The call for the provision of counselling and psychological support is supported by several studies.^22^ The provision of such services will help in curbing COVID-19-related burnout and stress. Another scholar^40^ further called for such measures as childcare, social assistance to the spouses and psychological support to protect the families and their future. The provision of transport for workers who test COVID-19 positive is commendable. The implementation of this recommendation would leverage on the already existing transport system and reorganising it in the context of COVID-19. This will curb the further spread of COVID-19 and protect the travelling public as healthcare workers may have been exposed to COVID-19 patients. The management will have to ensure that the vehicles are regularly cleaned and fumigated in line with COVID-19 infection prevention and control protocols. Scholars^41^ urge national leaders to assume responsibility for frontline workers safety. A related study^40^ found that healthcare workers valued organisational support that valued their basic human needs: free meals, access to free parking, areas to rest and recover. Therefore, the researchers recommend the establishment of a psychosocial support unit. Its mandate would be to provide individualised differentiated psychosocial support and follow up of staff who fall sick or are isolated at home in a uniform manner. This is feasible because the support system can be provided by already employed psychologist and nurses trained in mental health.

## Conclusion

This study provides an in-depth analysis of the healthcare workers’ experiences after contracting COVID-19. The results of this study show that study participants lacked psychosocial support, experienced financial challenges as they incurred diagnostic and treatment costs. The findings of this study will be critical for health authorities, programmers and policymakers to facilitate planning and preparedness for pandemics. Healthcare workers play a critical role in the country’s healthcare system. Therefore, this calls for a systematic and collaborative approach in their support. The researchers recommend the setting up of a differentiated service delivery support system for healthcare workers to cater for their mental health and considering that a ‘one-size-fits-all’ approach to providing support is unlikely to be helpful. Another recommendation is that providing support and or post-traumatic stress counselling must be an integral part of the COVID-19 response. Therefore, the researchers call for more collaboration and consultation in setting up a sustainable support system for healthcare workers.

## Limitations of the study

The study has some limitations that need to be acknowledged. Firstly, the study was conducted during the early phase of pandemic, as of now, the situation might have changed. The same study may be replicated to individuals who suffered from COVID-19 at different phases of the pandemic. The study was limited only to healthcare providers in Bulawayo Province, Zimbabwe; hence, findings report only the experiences in this province. However, through literature and observation, the lived experiences of healthcare providers in different settings in the sub-Saharan Africa are similar.

## Implications of the study

This study provides early evidence on the challenges faced by healthcare workers after contracting COVID-19. This information is of great interest to the policymakers, programme planners and health facility managers involved in the response to COVID-19 or any future pandemic. Differentiated comprehensive psychosocial support should be provided to enhance the well-being of healthcare providers.
